# Physical and psychoeducation combined group intervention: a quasi-experimental study with Portuguese cancer survivors

**DOI:** 10.1192/j.eurpsy.2023.235

**Published:** 2023-07-19

**Authors:** A. Torres, A. Ribeiro, C. Matos, J. Costa, A. F. Oliveira, I. M. Santos, S. R. Costa

**Affiliations:** 1 Department of Education and Psuchology, Center for Health Technology and Services Research (CINTESIS@RISE), Aveiro; 2 Department of Psychology and Education, University of Beira Interior, Covilhã; 3 Portuguese Association of Leukemias and Lymphomas, Porto; 4 Coimbra Health School, Polytechnic Institute of Coimbra, Coimbra; 5 Department of Education and Psychology, Center for Health Technology and Services Research (CINTESIS@RISE); 6 Department of Education and Psychology, William James Center for Research (WJCR), Aveiro; 7 Department of Education and Psychology, University of Trás-os-Montes and Alto Douro, Vila Real, Portugal

## Abstract

**Introduction:**

Cancer is a major public health problem worldwide and the risk of death from cancer has decreased continuously since 1991, therefore, This translates into an increasing number of cancer survivors (CS) worldwide.

During the survivorship seasons, CS face several short-term, long-term, persistent, and late-emerging health and psychosocial problems, including cancer-related pain, fatigue, menopausal symptoms, anxiety, depression, distress associated with the risk of cancer recurrence, chronic uncertainty, social disruption, alterations of sleep, sexual and cognitive dysfunctions.

Since 2002 that some researchers and clinicians argued that it is important to de-velop and implement rehabilitation programs for cancer patients that integrate both psychosocial and physical rehabilitation.

**Objectives:**

With the scarcity of studies on the effectiveness of combined interventions in this population, despite the strong recommendation to perform and study it, and aiming to contribute to a greater knowledge on the theme, the present work aims to build, implement, and evaluate a combined intervention program, which integrates psychoeducational intervention with physical exercise to cancer survivors and relatives, through the following indicators: psychopathological symptoms (anxiety and depression), self-concept, coping strategies, personal growth and QoL.

**Methods:**

A non-probabilistic convenience sample of 70 cancer survivors was assigned to: control (without intervention: n=32), combined intervention (n=21) and psychoedu-cation intervention (n=17) groups. Both intervention groups were 9 consecutive weeks duration. The combined intervention group benefited from 2 weekly exercise sessions additionally. It was administered before and after intervention the following questionnaires: demographic; Hospital Anxiety and Depression Scale (HADS); Clinical Self-concept Inventory (ICAC); Cancer Coping Questionnaire (CCQ); sub-scale of Personal Growth of the Psychological Well-being Scale (EBEP) and the World Health Organization Quality of Life Questionnaire (WHOQOL-Bref ).

**Results:**

It was observed a statistically significant reduction of anxiety and depression symptoms from the beginning to the end of the intervention, as well as a significative improvement of overall and all do-mains of self-concept and personal growth. It was not observed a significative difference on quality of life.

**Conclusions:**

The findings of this study contribute to support of the beneficial effect of combined intervention on psychological functioning of cancer survivors. Positive effects of the psychological program were observed but not into the same extent as in the combined intervention.

**Disclosure of Interest:**

None Declared

